# Supplementary material for: Complexome profiling of the Chlamydomonas psb28 mutant reveals TEF5 as an early PSII assembly factor
Source: Plant Cell. 2025 Mar 17;37(6):koaf055. doi: 10.1093/plcell/koaf055 (PMC12164590; doi:10.1093/plcell/koaf055)
Supplement: koaf055_Supplementary_Data [file koaf055_supplementary_data.zip › psb28_interactive.zip!/plots/intensity/qualityPlots/peptides/_Cre15.g635200.t1.2.html]

### Cre15.g635200.t1.2

Trivial name:   
SeqCoverage [median]: 21.00  
Mass: 17.08 kDa  
  
 

| Sequence | MissedCleavage | Mass [Da] | wtIntensity | muIntensity | Score |
| --- | --- | --- | --- | --- | --- |
| GLTEDAIVR | 0 | 972.52 | 0.00 | 303.18 | 55.68 |
| LLQEQPGFR | 0 | 1086.58 | 0.00 | 1219.89 | 116.37 |
| SIFQTGSAEEVEAAR | 0 | 1593.76 | 0.00 | 663.22 | 68.68 |
|  |  |  | 0.0 | 2186.3 |  |
